# Notes and News

**Published:** 1902-08-09

**Authors:** 


### Notes and News.

The Queen paid a visit to Netley Hospital on Monday. Since her last visit there the interior of the hospital has been much brightened. The corridors and wards are now hung with pictures, and the tables in the wards covered with bright tablecloths, and new beds have been provided, all the gift of Mr. W. H. Bischoffsheim.

A new operating theatre is being built at the Llanelly Hospital at a cost of £1,000.

A new pavilion has been opened at the Isolation Hospital, Aughton, at the cost of about £2,000. It is intended for the reception of scarlet fever cases, men, women and children.

Mr. Chamberlain has given £50 towards the funds of Charing Cross Hospital as a thankoffering for the care and attention which he received there when suffering from his recent accident.

The Glamorgan County Council have with the sanction of the Home Office passed by-laws forbidding spitting in public carriages, waiting-rooms, halls, and places of entertainment. The penalty is a fine not exceeding £5.

A convalescent home for children has been opened in connection with the Birmingham Hospital Saturday Fund at Great Barr, near Birmingham. The house is an old one renovated for the purpose, and will contain fifty children.

A fine sanatorium has been opened for consumptive patients of the richer classes amongst the Ochill Hills, near Kinross. The sanatorium provides 60 bedrooms, all with a south aspect. The public rooms consist of dining, drawing, writing, and music rooms, and library. In connection with the medical treatment there is an inhalation room, a Roentgen ray installation, and an induction coil arc lamp for ultra violet light, and spray, plunge, and Sitz baths. The grounds consist of upwards of 400 acres. The cost of the building was £35,000, defrayed by a company formed for the purpose by Sir Thomas Coats.

Sir F. Treves has been appointed Sergeant-Surgeon in Ordinary to the King.

Sir William Taylor is endeavouring to establish a journal devoted to the interests of the Army Medical Service.

The Belgian Surgical Society is inviting leading foreign surgeons to attend its Congress at Brussels this year, the dates being from the 8th to the 11th of September.

Captain Rost, of the Indian Medical Service, has been making experiments tending to show that the disease of beri-beri is connected with the consumption of fermented rice.

Sir Charles Seeley has presented the Old Children's Hospital at Nottingham to the General Hospital in the same town to be used as an isolation department. He attaches the condition that the iron building now used at the General Hospital for isolation cases shall be removed, and the space left free for all time.

Adulteration of food has reached a fine art at Camberwell. The Public Analyst of the Borough reports that nearly 16½ per cent. of the samples analysed by him were adulterated. This high percentage, he states, has been maintained for some years, so that the measures adopted to suppress the evil are evidently not of a deterrent character.

The water supply from the Southwark and Lambeth Companies has been called into question. There has been a good deal of typhoid at Wandsworth, and the Borough Council thinks it necessary to call the attention of the Local Government Board to the amount of organic matter shown by the Government Analyst to exist in the water supplied to them by the companies named.

The Middlesex Hospital is pursuing its valuable work in the crusade against cancer with vigour. Towards an annual sum of £2,000 required to carry out the complete scheme desired, £700 has already been subscribed, principally through the advocacy of Mr. Pearce Gould, surgeon to the hospital. The Drapers' Company are also contributing £50 per annum to assist the scheme of research. Besides the 45 beds available in the cancer wing, a large number of less advanced cases are treated in the general wards, and in the out-patients' department.

A new isolation hospital has been opened for the Isle of Thanet near Ramsgate. The building stands on a site consisting of 10 acres. The administration block is connected with the ward pavilions by open corridors. The accommodation is for 82 patients, beds being provided for 52 scarlet fever cases, 14 typhoid, 14 diphtheria, and two observation cases. The heating is by open fireplaces in the centre of the wards, supplemented by hot-air radiators. The ventilation is by windows and Boyle's self-acting ventilators. The cost of the buildings has been £49,500. The architects are Messrs. Langham and Cole, Ramsgate.

